# Assessing ultraviolet-C light-emitting diode disinfection of disposable video laryngoscope blades: a sustainable approach through an integrated microbiological, environmental, economic, and regulatory evaluation

**DOI:** 10.1016/j.bjao.2026.100566

**Published:** 2026-05-28

**Authors:** Hannah Siwe, Bjorn Delbeecke, Piet Cools, Philip Meuleman, Pascal Verdonck, Alain F. Kalmar

**Affiliations:** 1 Laboratory of Liver Infectious Diseases, Department of Diagnostic Sciences, Faculty of Medicine and Health Sciences, Ghent University, Ghent, Belgium; 2 Research and Development, eLEDricity, Merelbeke, Belgium; 3 Department of Electronics and Information Systems, IBiTech, Ghent University, Ghent, Belgium; 4 Laboratory Bacteriology Research, Department of Diagnostic Sciences, Faculty of Medicine and Health Sciences, Ghent University, Ghent, Belgium; 5 Department of Anaesthesia, Intensive Care and Pain Medicine, General Hospital Maria Middelares, Ghent, Belgium

**Keywords:** high-level disinfection, lifecycle analysis, light-emitting-diodes, sustainability, ultraviolet-C, video laryngoscope blades

## Abstract

**Background:**

The shift from reusable to single-use anaesthesia devices, such as laryngoscope blades, is increasingly questioned because of the financial and environmental costs of disposables. Validated disinfection methods enabling safe reuse could provide clinical, economic, and sustainability benefits.

**Methods:**

In this laboratory-based experimental study, disposable McGRATH™ X-blade video laryngoscope blades were artificially contaminated with *Staphylococcus aureus* and subjected to automated ultraviolet-C (UV-C) light-emitting diode (LED) disinfection under different reprocessing conditions. An integrated assessment was performed, including microbiological efficacy, material integrity after repeated UV-C exposure, estimated economic and environmental impact, and regulatory considerations. Results were compared with single-use, steam autoclaving, and chlorine dioxide wipe-based disinfection.

**Results:**

UV-C disinfection reduced *Staphylococcus aureus* contamination to undetectable levels across all experimental conditions; unpackaged cleaned, uncleaned blades, and packaged uncleaned blades (*n*=6, *n*=5, and *n*=5, respectively). No visible degradation, functional impairment, or deterioration in image quality was observed after repeated UV-C exposure. Per-cycle cost was less than €0.20, and greenhouse-gas emissions were reduced by 92–94% compared with single-use. In contrast, autoclaving or chemical disinfection compromised blade usability.

**Conclusions:**

Automated UV-C LED treatment effectively disinfected McGRATH X-blades under controlled laboratory conditions, preserving material integrity and offering substantial environmental and economic advantages. This approach may support more sustainable anaesthetic practice where validated point-of-care reprocessing is implemented.

**Editor’s key points**

• Validated disinfection methods enabling the safe reuse of single-use video laryngoscope blades could provide clinical, economic, and sustainability benefits.
• This laboratory-based investigation assessed the feasibility of ultraviolet-C light-emitting diodes (UV-C LED) disinfection for the reprocessing of single-use video laryngoscope blades.
• Automated UV-C LED treatment effectively disinfected McGRATH™ video laryngoscope blades under controlled laboratory conditions, preserving material integrity and offering substantial environmental and economic advantages.
• UV-C LED high-level disinfection may enable a much needed solution for single-use devices of various consumables in anaesthesia, such as laryngoscope blades, offering a cost-effective alternative while also reducing the environmental impact of healthcare practices.

Health care systems significantly contribute to environmental pollution, adversely affecting human health.^1^^,^^2^ The sector contributes to global warming and climate change through water, air, and soil contamination, as well as through substantial waste generation.^2^ Sustainable health care seeks to provide medical services in an environmentally responsible manner, encompassing strategies such as replacing harmful chemicals with safer alternatives, minimising water use and waste production, and preserving natural resources.^1^^,^^3^ Medical device reprocessing represents an area where sustainability principles are particularly relevant.

The World Health Organization defines medical device reprocessing as the procedures required to prepare a contaminated reusable device for its intended use, which may encompass different steps including cleaning, packaging, labelling, disinfection and sterilisation.^4^ Laryngoscopes are semi-critical items requiring at least high-level disinfection (HLD), which should eliminate all forms of microbial life except for large numbers of bacterial spores and prions.^5^, ^6^, ^7^ HLD should be preceded by thorough cleaning to remove (in)organic material to maximise the efficacy of this procedure.^4^^,^^6^ Reprocessing of reusable laryngoscope blades is therefore a time and resource-intensive process, particularly when using moist heat sterilisation carried out in a central sterilisation department.^8^ This process requires significant water consumption, even when idle, and is limited to heat-resistant objects, restricting its applicability.^4^^,^^9^^,^^10^ As an alternative, chlorine dioxide wipes can be used in a decentralised manner at the point of care. In this context, decentralised refers to use within the peri-operative environment, operated by trained personnel following validated protocols. These wipes are impregnated with chemicals, posing risks to patients, personnel, and the environment while involving a series of manual steps that require strict adherence to the manufacturer’s specified contact time and application method to ensure effective disinfection.^4^^,^^10^, ^11^, ^12^ The manual nature of this process remains time-consuming and labour-intensive and may result in inconsistent disinfection due to human error, underscoring the limitations of this approach.^13^^,^^14^

Despite reusable options available, many traditional surgical tools have transitioned to single-use devices (SUDs).^15^ Since the mass production of plastics in the 1950s, the hospital supply industry began replacing traditional materials such as glass, metal and rubber with polymers.^16^^,^^17^ Manufacturers adopted sterilisation techniques based on ethylene oxide and gamma radiation, which are not readily available in hospitals, to accommodate disinfection of these new materials.^17^^,^^18^ This resulted in a shift towards prepackaged, presterilised devices labelled as ‘single use only’, partly motivated by the commercial interests and recommendations based on the ability to support safe reuse.^15^^,^^17^^,^^19^^,^^20^ A shift in responsibility for patient safety due to transmission concerns associated with reprocessing, alongside other advantages including improved time efficiency, and lower upfront costs, further supported the move from reusables to SUDs in health care.^20^, ^21^, ^22^ However, to address health care waste and limit resource depletion, transitioning from single-use products to reusables or reusing SUDs represents a rational and effective strategy.^3^^,^^15^

To support the reprocessing of SUDs, ultraviolet-C (UV-C) disinfection may provide a valuable solution. Interest in the use of UV-C has significantly increased since the coronavirus disease 2019 pandemic, with UV-C light-emitting diodes (LEDs) emerging as a more sustainable alternative compared with conventional low-pressure mercury vapour lamps.^23^, ^24^, ^25^, ^26^ UV-C disinfection chambers, like manual wipe systems, allow for decentralised use and do not rely on water, chemicals or heat, and have low energy consumption.^27^ Unlike manual wipes, UV-C devices are automated, which is recommended when feasible, and enables full traceability of each disinfection cycle, including device identification and delivered dose (mJ cm^−2^), which can be linked to the hospital information system.^28^

A potential use case for UV-C disinfection is single-use McGRATH™ video laryngoscope blades made from a transparent medical-grade polymer.^29^ Video laryngoscope blades were selected because their heat- and chemical-sensitive polymer construction limits the applicability of conventional reprocessing methods, and provides a relevant model to assess whether UV-C LED can achieve effective microbial inactivation without compromising material integrity. This study aimed to evaluate automated UV-C LED disinfection for the reprocessing of video laryngoscope blades, assessing disinfection efficacy, impact on material integrity and image quality, economic cost, environmental impact (CO_2_ emissions), and regulatory considerations in comparison with autoclave sterilisation, chlorine dioxide disinfection, and single-use.

## Methods

### Study design

The study was designed as a laboratory-based investigation, assessing the feasibility of UV-C LED disinfection of single-use video laryngoscope blades. The implications of reuse were evaluated in comparison with disposable use, autoclave sterilisation and chlorine dioxide disinfection. No ethical approval was required as no human participants were involved.

### Microbiological assessment

#### Bacterial suspension

*Staphylococcus aureus* (American Type Culture Collection 25923) stored at −80°C was plated onto tryptic soy agar supplemented with 5% sheep blood (TSA-5%SB) (BD, Franklin Lakes, NJ) using the quadrant streak method.^30^ After 18–24 h at 37°C, a single colony was passaged onto TSA-5%SB using the continuous streak method with a 10 μl loop and incubated for another 18–24 h. A bacterial suspension was freshly prepared using a 10 μl loop to collect *S. aureus* colonies from the TSA-5%SB plate, which were then resuspended in sterile NaCl 0.9% solution (saline) to an optical density (600 nm) between 1.0 and 1.7, equivalent to a concentration of approximately 10^9^ CFU ml^−1^.

#### Contamination and UV-C treatment

**Fig 1:**
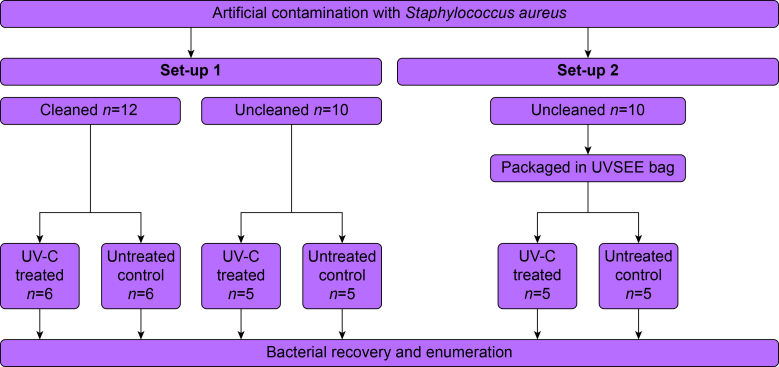
Schematic overview of the microbiological assessment. Laryngoscope blades were artificially contaminated and subsequently subjected to defined reprocessing sequences, including cleaning, packaging in a UVSEE bag, and ultraviolet-C (UV-C) disinfection, with corresponding untreated controls handled in parallel, followed by bacterial recovery and enumeration to determine the log10 reduction. Numbers indicate the sample size for each condition. No samples were excluded from analysis.

McGRATH X-Blade size 3 single-use laryngoscope blades (REF: X3-003-000, Medtronic, Dublin, Ireland) (Supplementary Fig. 1) were partially submerged in pairs in 100 ml of the bacterial suspension in a 100 ml multipurpose beaker, with the hollow insert for the camera facing upwards. The capacity of the beaker exceeded 100 ml, with no risk for spillage. The submerged blades were then placed at room temperature for 10 min. After removal of the blades, excess droplets were removed by tapping the instrument four times against the beaker. Immediately after artificial contamination, blades were subjected to different reprocessing protocols, defined here as a sequence of cleaning, packaging and disinfection steps (Fig. 1). Cleaning consisted of holding the blades under a continuous stream of tap water for 3 s, after which excess droplets were removed by gently dabbing the instrument with tissue paper. Packaging consisted of placing the blade inside a UVSEE™ bag (eLEDricity, Merelbeke, Belgium), which has no disinfecting function and is used solely to allow sealed handling and transport after UV-C treatment. Disinfection specifically refers to UV-C treatment inside the disinfection chamber. Two experimental setups were evaluated. In the first setup, blades were used without a UVSEE bag. Both cleaned and uncleaned blades were exposed to UV-C and compared with corresponding cleaned and uncleaned blades that did not undergo UV-C treatment. In the second setup, uncleaned blades were placed inside a UVSEE bag prior to UV-C exposure and compared with uncleaned, bagged blades that did not undergo UV-C treatment. In each setup, untreated blades served as the respective controls to assess the microbial reduction.

For UV-C treatment, laryngoscope blades were placed in a ZAPARAY™ UV-C LED disinfection prototype (eLEDricity) (Supplementary Fig. 2). This device is an enclosed radiation chamber with a pull-out drawer featuring a quartz bottom, transparent to UV-C. Equipped with UV-C LEDs at the top and bottom, the chamber delivered a UV-C dose of 118 mJ cm^−2^ centrally in the drawer in a 5-min cycle.

#### Bacterial recovery and enumeration

To recover bacteria, each blade was placed in an empty 100 ml multipurpose beaker with the hollow insert for the camera facing upwards and rinsed with 10 ml saline. This procedure was repeated a total of five times using the same 10 ml volume. Subsequently, the 10 ml fluid was collected in a 15 ml tube and vortexed. Bacterial enumeration of the collected fluid was performed as described previously.^26^ Briefly, a tenfold dilution series of the collected fluids was prepared, and a 5 μl droplet of each dilution was plated onto TSA-5%SB. Plates were incubated for 18–24 h at 37°C, after which colonies were counted with the naked eye. The dilution yielding the highest colony count between 0 and 100 was used to calculate the concentration, expressed in colony-forming units (CFU) ml^−1^. The log_10_ reduction was calculated with the following formula: log_10_ (CFU ml untreated control – CFU ml^−1^ treated).

To assess residual bacteria on the blades after bacterial recovery with 10 ml saline, blades were imprinted onto TSA-5%SB, tracing their curvature. Imprinted plates were incubated for 18–24 h at 37°C and examined for bacterial growth.

#### Statistical analysis and data visualisation

GraphPad Prism software version 10.4.1 was used for data visualisation. Descriptive statistics were used to summarise untreated control concentrations (CFU ml^−1^) as median and interquartile range, and log_10_ reductions as mean and standard deviation. Mean log_10_ reductions were compared across the three reprocessing protocols using one-way analysis of variance (anova), followed by Tukey’s multiple-comparison test to identify pairwise differences. A *P*-value<0.05 was considered statistically significant.

### Environmental assessment

A comparative, attributional life cycle assessment (LCA) was performed to quantify and compare the climate change impact of four scenarios: single-use, steam autoclave sterilisation, manual chlorine dioxide wipe-based disinfection, and UV-C LED disinfection, all within the same hospital setting and using the European electricity grid carbon intensity. The analysis was restricted to the climate change impact category, with results expressed as greenhouse-gas emissions in carbon dioxide equivalents (CO_2_e). The comparison was intended as an order-of-magnitude benchmark, rather than to imply direct equivalence between scenarios. To assess the environmental impact, an LCA was conducted of UV-C disinfection, manual disinfection and single-use, using the openLCA software (GreenDelta, Ltd, Berlin, Germany) with the ELCD (European reference Life Cycle Database of Joint Research Centre, Version 3.2 from October 2015) and BIOENERGIEDAT database. The LCAs were performed with one laryngoscope blade use as the functional unit for modelling. We compared the environmental impact of single use of both an X-blade and a Blade 4 McGRATH disposable blade with reuse through two different reprocessing methods: (1) UV-C disinfection using the RAY-TWO™ device, with a single blade per disinfection cycle, combined with 500 ml of rinsing water and a paper towel per cycle, and (2) conventional manual point-of-care HLD using Tristel™ Trio Wipes.

For autoclaving, emission data and per-cycle costs were sourced from a recent LCA of reusable flexible laryngoscopes that modelled steam sterilisation in a hospital setting.^31^ Although the flexible laryngoscopes and video laryngoscope blades differ in geometry and mass, autoclave energy and water consumption are largely determined by fixed cycle parameters rather than marginal load differences, rendering this comparison conservative. With the autoclave typically run with five laryngoscopes,the estimated emission was 330 g CO_2_ per item sterilised (adjusted for EU-27 average electricity carbon intensity of 213 g CO_2_e kWh^−1^).^31^, ^32^ While autoclaving is not technically suitable for the polymer video laryngoscope blades studied here, it was included as a comparator because steam sterilisation remains one of the most widely used hospital reprocessing methods and therefore provides a relevant environmental benchmark.

For each scenario, the LCA included all consumables, energy and water inputs directly associated with a single laryngoscope blade use, as described in the economic assessment. This comprised device-specific materials, reprocessing-related consumables (e.g. wipes, gloves, paper towels, packaging), and energy and water use associated with disinfection or sterilisation. Environmental impacts were modelled at the level of individual components and process steps but are presented as aggregated scenario-level outcomes for comparative purposes, as shown in Table 1.

**Table 1:** CO_2_ emissions and cost associated with three scenarios. Single-use (for both the X-blade and Blade 4 McGRATH video laryngoscopy blades); high-level disinfection via autoclave; manual point-of-care disinfection with Tristel Trio wipes; UV-C disinfection (including 500 ml rinsing water and paper towel), and UVSEE bag alone. Reported values represent the aggregated environmental and economic impact of all modelled components within each scenario. Values represent deterministic model-based estimates per functional unit; as such, no variability measures or sample sizes apply. The functional unit for modelling was one laryngoscope blade, and assuming 50 000 cycles for the UV-C. Autoclave values sourced from Kidane and colleagues^31^; costs converted to € at January 2026 exchange rate. CO_2_e adjusted for the EU-27 average electricity carbon intensity of 213 g CO_2_e kWh^−1^.^32^ CO_2_e, carbon dioxide equivalent; EU, European Union; UV-C, ultraviolet-C.

	Single use		Autoclave	Point-of-care Tristel	Point-of-care UV-C	
	X-blade	Mac 4			Disinfection	UVSEE bag
CO_2_ emissions (g)	92.40	131.80	330.00	120.50	7.50	11.40
Cost (€)	24.00	6.30	1.60	7.05	0.101	0.60

In the reuse scenarios, only the disinfection-related emissions were modelled, as the production of the initially manufactured blade represents a preexisting burden. In the single-use scenario, cradle-to-grave emissions, including production and disposal, were fully accounted for. Reuse was therefore modelled as the marginal replacement of additional single-use blades. For single-use blades, the reported CO_2_e values represent cradle-to-grave emissions, including manufacturing, packaging, and end-of-life disposal via medical waste incineration, consistent with published life cycle assessments.^8^ The emissions of the UVSEE bag were calculated separately. For UV-C, equipment replacement was assumed after 50 000 cycles. LCA modelling consolidated various material and energy inputs, along with pollutant emissions, into several key environmental impact categories. In our analysis, the primary category of interest was the contribution to global warming from greenhouse-gas emissions, measured in CO_2_e.

### Economic assessment

Calculations were based on the prices excluding the value added tax listed on official supplier websites as the actual price of medical devices may vary depending on hospital tenders. For electricity usage, a wholesale price of €0.15 kWh^−1^ was used. The cost of the Tristel Trio wipes and nitrile gloves were assumed €450 per box of 50 units and €5.00 per box of 200 units, respectively. The RAY-TWO UV-C device was assumed to cost €5000 with an electricity consumption of 0.005 kWh, or €0.00076 per cycle. For cleaning, 0.5 L of rinsing tap water and one paper towel per cycle were assumed. UVSEE bags were assumed €0.6 per bag. The autoclave cost and CO_2_e per cycle were sourced from Kidane and colleagues.^31^ CO_2_e values were adjusted for the EU-27 average electricity grid carbon intensity of 213 g CO_2_e kWh^−1^.^32^ More recent estimates indicate a reduction of approximately 10% in EU electricity carbon intensity since the time of modelling. As electricity contributes both directly (e.g. autoclaving, UV-C operation) and indirectly through upstream processes such as device manufacturing, disposable production, paper towel fabrication, and water treatment, progressive grid decarbonisation would proportionally reduce emissions across all scenarios, including the amortised footprint of the UV-C device, without materially altering their relative ranking. In all scenarios, only supplies and equipment were considered, while logistical handling, labour time, and transportation costs were excluded due to their speculative nature.

### Impact on physical integrity and image quality

Both the McGRATH video laryngoscope and blades underwent 500 UV-C disinfection cycles (equalling 50 J cm^−2^) to evaluate integrity and image quality. Physical integrity was assessed via discolouration and through an applied force of 98 N to new and irradiated blade tips. Five anaesthesiologists performed a blinded comparison of image quality, as a proxy for lens coating opacity and clinical usability, across non-irradiated, irradiated, chlorine dioxide treated and autoclaved blades for clinical significance.

### Legal assessment

The analysis primarily focused on the Medical Device Regulation (MDR) 2017/745 which applies to countries of the European Union (EU) and the European Economic Area, to determine the conditions under which SUDs may be reprocessed. As a starting point, the MDR itself was examined, with particular attention to the use of the term ‘single-use’ to identify the relevant provisions and guidance. Additionally, national legislation governing the reprocessing of SUDs were systematically reviewed to capture country-specific approaches and differences across the EU and the European Economic Area.

## Results

### Microbiological assessment

**Fig 2:**
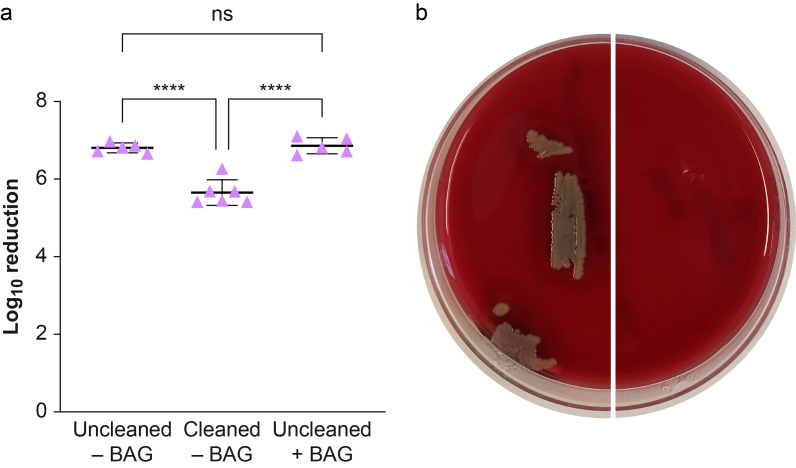
Microbiological efficacy of UV-C treatment on McGRATH X-blade laryngoscope blades. (a) Individual data points represent the log10 reductions obtained under different reprocessing protocols: uncleaned without packaging in a UVSEE bag (*n*=5, three independent experiments), cleaned without packaging in a UVSEE bag (*n*=6, three independent experiments), and uncleaned and packaged in a UVSEE bag (*n*=5, three independent experiments). Read-out was performed after 18–24 h incubation. Horizontal lines indicate the mean with error bars representing the standard deviation. Statistical analysis was performed using one-way anova followed by Tukey’s multiple-comparison test; ∗∗∗∗ indicates *P*<0.0001, and ns indicates not significant. (b) Bacterial growth observed of instrument imprints of uncleaned laryngoscope blades in the absence of a UVSEE bag, with the left side representing the untreated instrument, and the right side representing the treated instrument. Read-out was performed after 18–24 h incubation. anova, analysis of variance.

The microbiological assessment revealed that the median concentration of untreated controls was 6.80×10^6^ (4.90×10^6^–8.50×10^6^) CFU ml^−1^ for the uncleaned group and 3.80×10^5^ (2.60×10^5^–8.40×10^5^) CFU ml^−1^ for the cleaned group without a UVSEE bag, and 6.60×10^6^ (4.70×10^6^–1.19×10^7^) CFU ml^−1^ for the uncleaned group with a UVSEE bag. No bacteria were recovered after UV-C treatment in all cases, resulting in a mean standard deviation log_10_ reduction of 6.81 (0.13), 5.65 (0.33), and 6.86 (0.21), respectively (one-way anova, *P*<0.0001) (Fig. 2a). Tukey’s multiple comparisons test showed that the cleaned group without a UVSEE bag differed significantly from both the uncleaned group without a UVSEE bag (mean difference 1.16, 95% confidence interval [CI]: 0.77–1.55, *P*<0.0001) and the uncleaned group with a UVSEE bag (mean difference −1.21, 95% CI: −1.60 to −0.82, *P*<0.0001), whereas the two uncleaned groups did not differ significantly (mean difference −0.05, 95% CI 0.46 to 0.36, *P*=0.946), indicating that the observed differences in log reduction were attributable to the cleaning step. The complete absence of *S. aureus* in each of the three scenarios was further confirmed with the imprint of the laryngoscope blades on TSA-5%SB, showing no bacterial growth for the treated blades (Fig. 2b, Supplementary Fig. 3).

### Environmental and economic assessment

The results of the environmental and economic assessment are shown in Table 1. The cradle-to-grave carbon emissions (including waste incineration) of one single-use X-blade (14.7 g plastic) and Mac 4 (20.9 g plastic), including packaging, were 92.4 g CO_2_e and 131.8 g CO_2_e, respectively. A set of Tristel Trio wipes and activator foam for a single disinfection, including the cardboard and individual packaging, resulted in 69.3 g CO_2_e. One pair of nitrile gloves added 51.2 g CO_2_e, bringing the total to 120.5 g CO_2_e.^33^ One UVSEE bag 100 mm × 150 mm (1.86 g plastic) represents 11.4 g CO_2_e.

The RAY-TWO LCA for 50,000 cycles showed 73 kg CO_2_e for the device itself, 215 kg CO_2_e for 50 000 paper towels, 35 kg CO_2_e for 25 000 L of rinsing water, and 53.9 kg CO_2_e for 253 kWh of electricity consumption using the EU-27 average carbon intensity of 213 g CO_2_e kWh^-1^.^32^ Altogether, this amounted to 376.9 kg CO_2_e for 50 000 cycles, or 7.5 g CO_2_e per cycle. Of this total, the energy-related emissions of the UV-C treatment represented 1.08 g CO_2_e.

### Impact on integrity and image quality

After 500 UV-C disinfection cycles applied to both the McGRATH video laryngoscope camera unit and blades, no visible degradation, functional impairment, or image quality deterioration was observed, including blurring, discolouration*,* or reduced brightness. In contrast, a single autoclave cycle or disinfection with chlorine dioxide wipes rendered the blades unusable due to blurring of the viewing window.

### Legal assessment

According to Article 17(§1) of the MDR, reprocessing of single-use laryngoscope blades is only allowed when permitted by national law. The reprocessor, often the hospital, is considered the manufacturer and assumes primary responsibility, including implementing a quality management system, validating processes, and ensuring that the safety and performance of the reprocessed blade are equivalent to the original.^34^ However, member states differ in their approach, as a total of 18 countries currently prohibit all reprocessing of SUDs, including France, Italy and Finland, while other countries, such as Belgium, Germany, the Netherlands and Sweden, permit reprocessing under strict conditions.^35^ In countries where it is allowed, anaesthetists must follow hospital protocols. It is the hospital’s duty to ensure that validated procedures are in place and compliant with national and EU-level safety standards. If there are no validated protocols available, anaesthetists must assume that reprocessing of SUDs is not legally permissible.

In contrast, reprocessing of reusable devices is regulated to maintain device performance throughout multiple cycles of use. Annex I, Chapter II, Section 23.4 (n) of the MDR states that manufacturers of reusable instruments and other devices must provide validated instructions for appropriate processes that allow reuse, including cleaning, disinfection, packaging, and, where appropriate, validated methods of resterilisation suitable for the Member State or States in which the device is marketed. Information must also be provided to help users identify when the device should no longer be reused, such as signs of material degradation or the maximum allowable number of reuses.^34^ Detailed reprocessing instructions imply specifying the validated methodology in detail, rather than offering generic guidance. This suggests that manufacturers must clearly indicate the appropriate method along with necessary parameters, including time or concentration, which ensures users can reliably and safely carry out reprocessing as intended throughout its reusable lifespan.

Regulatory approaches to reprocessing of SUDs vary substantially across jurisdictions; in some countries reprocessing is prohibited, whereas in others, it is permitted under strict obligations. Consequently, implementation, generalisability, and feasibility depend on national law and appropriate regulatory approval.

## Discussion

Although environmental concerns are seldomly addressed, SUDs are receiving growing attention due to their significant environmental impact.^8^ Notably, the ‘single use’ designation is determined by the manufacturer, which reflects their commercial considerations and recommendations based on the ability to support safe reuse.^19^^,^^20^ In this regard, this study investigated the environmental and economic footprints of disposable use compared with conventional methods (autoclave and chlorine dioxide Tristel Trio wipe system) and UV-C LED. To further assess the feasibility of UV-C LED, image quality, physical integrity and microbiological efficacy were investigated.

The economic assessment revealed that reuse via UV-C LED disinfection of unbagged blades would reduce costs by 99.6% and 98.4% compared with single-use Blade 4 and X-blade, respectively. UV-C LED disinfection was also 93.6% and 98.6% less expensive than autoclaving and chlorine dioxide disinfection, respectively. Likewise, the environmental assessment showed that UV-C disinfection enabled a 92% and 94% reduction in CO_2_ emissions compared with disposable blades, a 98% reduction compared with autoclaving, and a 94% reduction compared with chlorine dioxide disinfection. All UV-C associated calculations were based on a disinfection procedure involving a single blade, whereas autoclave impacts were based on five items per run.^31^ However, in practice, more than one item could be placed in the UV-C LED device, which may result in more favourable values than those already presented. Additionally, differences in logistics and personnel costs were not considered, although it is reasonable to assume that these would be higher for high-volume use of SUDs, centralised autoclaving and manual disinfection, considering associated handling, distribution, and disposal. Furthermore, the social impacts associated with the implementation of UV-C LED disinfection were not assessed and fall outside the scope of the present study.

From a microbiological perspective, the UV-C LED chamber was able to effectively reduce the bacterial load to undetectable levels under controlled laboratory conditions, regardless of the reprocessing protocol used, ranging from 5.65 to 6.86 log_10_ reduction. The UVSEE bag had no negative impact on UV-C inactivation, indicating the feasibility for storage of disinfected devices, although the shelf life may warrant further investigation. Data indicated that rinsing alone reduced the bioburden by approximately 1 log, demonstrating the impact of cleaning in line with established guidelines.^6^^,^^7^

Although not the most prevalent oral commensal, *S. aureus* was used as a reference organism because of its clinical relevance and widespread use in standardised disinfection testing.^36^, ^37^, ^38^, ^39^ UV-C acts through non-species-specific photodamage targeting nucleic acids, proteins and lipids, with available literature demonstrating the broad efficacy of UV-C in inactivating microorganisms, including spores, suggesting that common oral bacteria are also susceptible.^40^, ^41^, ^42^, ^43^, ^44^ While this approach provides a reproducible assessment of disinfection efficacy, further studies in clinical settings may be warranted to confirm the UV-C dose and performance under real-world conditions involving mixed oral flora and organic soiling.

While this study focused on video laryngoscope blades made from medical polymers, UV-C LED disinfection is not inherently limited to this material. Comparable results have been demonstrated for stainless steel devices, but depend on shape and technical characteristics.^26^ Considering the findings from the present study, this suggests that reusable metal blades or video laryngoscopes may be equally well disinfected. Similarly, UV-C LED may be a solution for the handles, which do not directly contact the patient's oropharynx, but for which well-defined disinfection protocols are currently lacking, with contamination clearly demonstrated.^45^ A recent guideline reported strong expert consensus on the universal implementation of video laryngoscopy, as a result of several advantages associated with its use, including a lower risk of oesophageal intubation and decreased likelihood of hypoxemic events.^46^, ^47^, ^48^ Despite this, the higher cost remains a limiting factor in universal implementation.^49^ In this context, UV-C LED disinfection could offer a solution to reduce costs and ease logistics.

Furthermore, UV-C LED disinfection is a non-thermal process, generating negligible heat compared with conventional UV-C mercury, thereby eliminating the risk of heat-induced material degradation.^27^ The non-thermal nature allows treatment of electronics and battery-containing components, which would be incompatible with heat-based reprocessing methods such as autoclaving. However, UV-C disinfection is limited by line-of-sight exposure, and shadowed areas may not receive a sufficient UV-C dose. Consequently, the suitability of devices for UV-C LED disinfection must be evaluated, considering geometry, materials, and intended use.^26^ Given that manual wipe-based point-of-care disinfection may be challenging for complex objects and that centralised reprocessing often precludes electronic components, decentralised UV-C LED disinfection has the potential to reduce residual contamination of these devices compared with current practice.

Beyond technical feasibility, financial constraints, staff limitations or regulatory requirements ultimately determine whether such practices can be adopted in clinical settings, and may vary both between EU member states and across non-EU jurisdictions.^35^^,^^50^, ^51^, ^52^ Certain challenges inherent in contemporary healthcare may impede the integration of environmentally sustainable practices. These results have demonstrated that UV-C LED disinfection may enable an effective solution for HLD of various consumables in anaesthesia, such as laryngoscope blades, providing a cost-effective alternative while also reducing the environmental impact of healthcare practices.^53^

## Authors’ contributions

Conceptualisation: PV, AFK

Writing – original draft and formal analysis: HS, AFK

Writing – review & editing and investigation: HS, BD, AFK

Methodology: HS, BD, PC, PM, AFK

Resources and supervision: PC, PM, AFK

## Funding

Flanders Innovation and Entrepreneurship (VLAIO) (grant number HBC.2021.0849).

## Declarations of interests

The UVSEE bags are registered under patent number BE1032021. The main authors declare the following financial interests/personal relationships, which may be considered as potential competing interests: HS and BD are employees of eLEDricity, and AFK is a shareholder of eLEDricity. All other authors declare they have nothing to disclose.
