# Supplementary material for: Assessing ultraviolet-C light-emitting diode disinfection of disposable video laryngoscope blades: a sustainable approach through an integrated microbiological, environmental, economic, and regulatory evaluation
Source: BJA Open. 2026 May 28;18:100566. doi: 10.1016/j.bjao.2026.100566 (PMC13240735; doi:10.1016/j.bjao.2026.100566)
Supplement: multimedia component 1 [file mmc1.docx]

SUPPLEMENTARY DATA

**Ultraviolet-C light-emitting diode disinfection of video laryngoscope blades as a use case for sustainable practice in anaesthesia: integrated microbiological, environmental, economic and regulatory assessment**

**Hannah Siwe^1,2^,** Bjorn Delbeecke^2,3^, Piet Cools^4^, Philip Meuleman^1^, Pascal Verdonck^3^, **Alain F. Kalmar^3,5^**

1. Laboratory of Liver Infectious Diseases, Department of Diagnostic Sciences, Faculty of Medicine and Health Sciences, Ghent University, Ghent, Belgium
2. Research and Development, eLEDricity, Merelbeke, Belgium
3. Department of Electronics and Information Systems, IBiTech, Ghent University, Ghent, Belgium
4. Laboratory Bacteriology Research, Department of Diagnostic Sciences, Faculty of Medicine and Health Sciences, Ghent University, Ghent, Belgium.
5. Department of Anaesthesia, Intensive Care and Pain Medicine, General Hospital Maria Middelares, Ghent, Belgium.


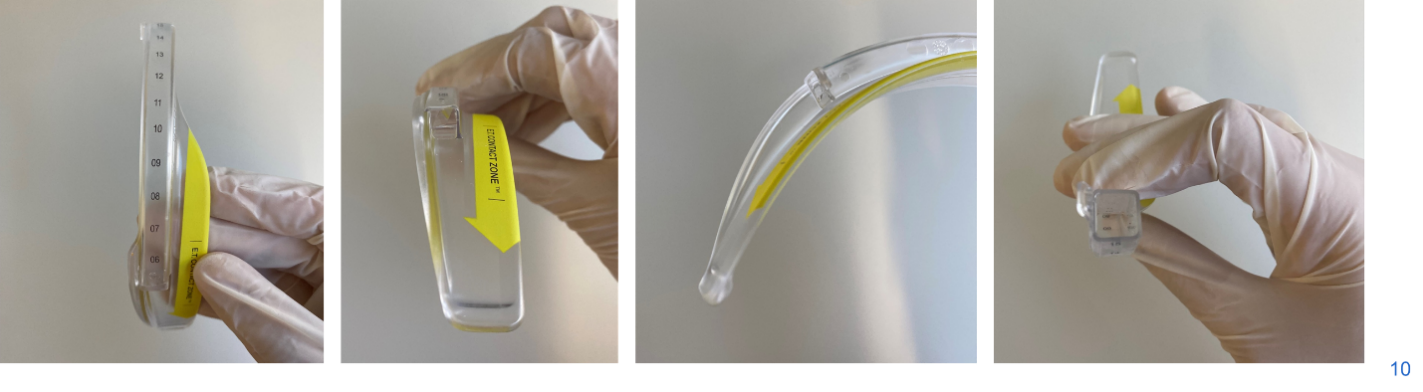


**Supplementary Figure 1: McGRATH X-blade laryngoscope blade.**

**
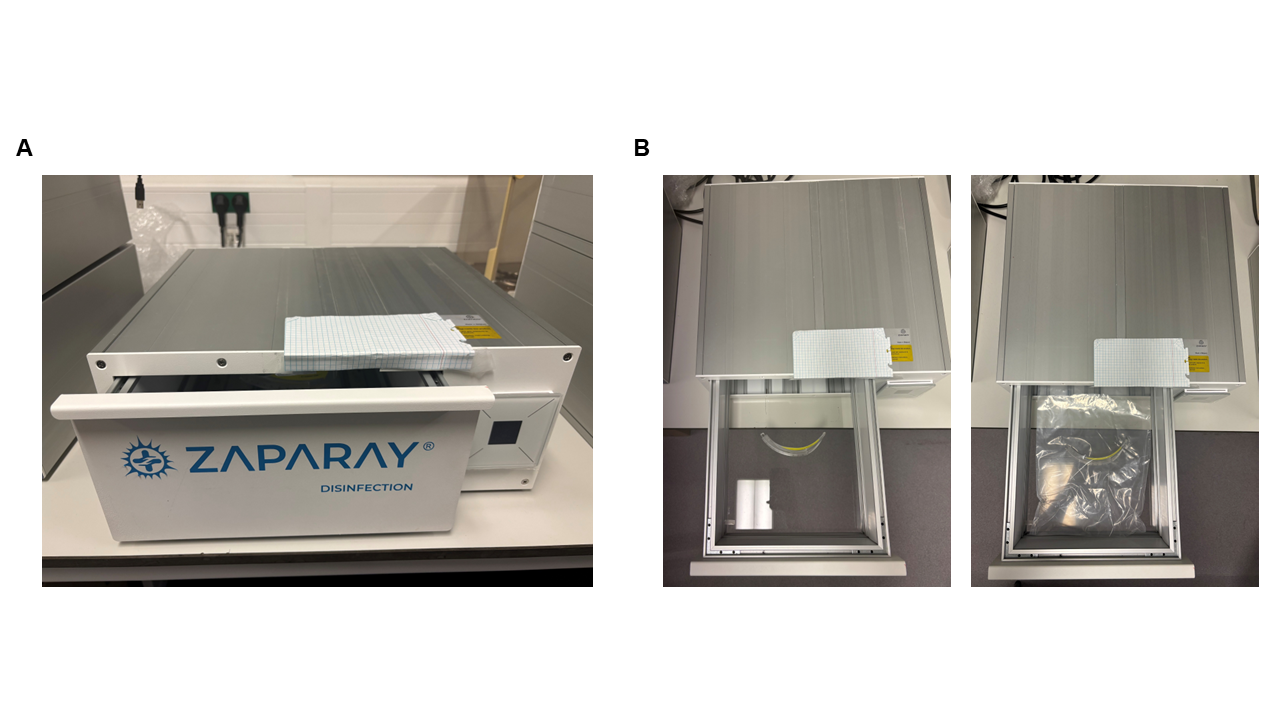
**

**Supplementary Figure 2: ZAPARAY UV-C LED disinfection prototype.** Test setup of the disinfection device showing (**A)** front view with the drawer partially opened, and **(B)** top view with the drawer extended, illustrating the laryngoscope blade unpackaged (left) and packaged in a UVSEE bag (right).


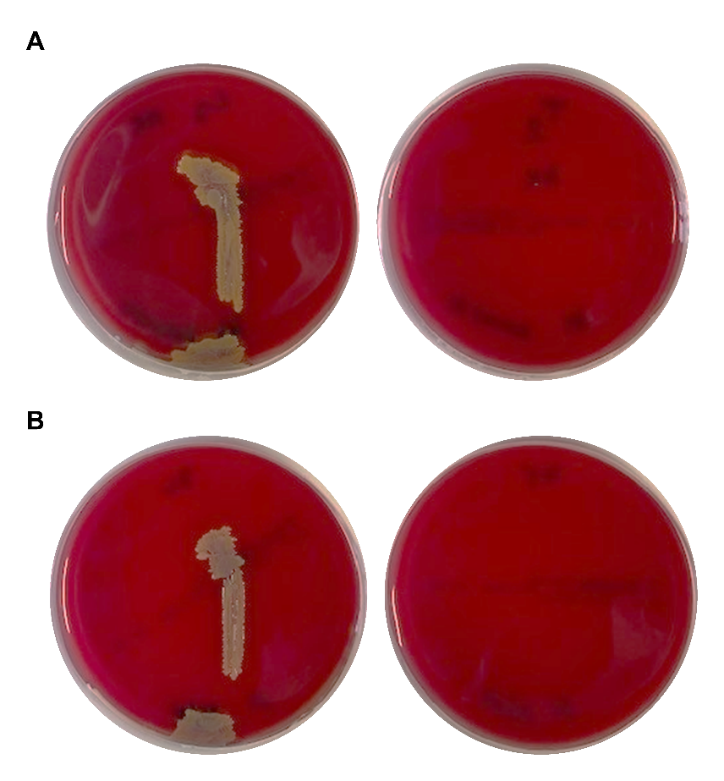


**Supplementary Figure 3:** **Instrument imprints of the McGRATH laryngoscope blades.** Bacterial growth observed of the instrument imprints on tryptic soy agar supplemented with 5% sheep blood. **A)** uncleaned laryngoscope blades packaged in a UVSEE bag, with untreated (left) and ultraviolet-C (UV-C) treated (right) imprints, **B)** unpackaged cleaned laryngoscope blades with untreated (left) and UV-C treated (right) imprints.
